# Forensic Dermatology of Postmortem Tanning: A Unique Phenomenon That Can Occur After Death

**DOI:** 10.7759/cureus.89375

**Published:** 2025-08-04

**Authors:** Philip R Cohen, Joseph A Prahlow

**Affiliations:** 1 Dermatology, University of California, Davis Medical Center, Sacramento, USA; 2 Dermatology, Touro University California College of Osteopathic Medicine, Vallejo, USA; 3 Maples Center for Forensic Medicine, Univeristy of Florida College of Medicine, Gainesville, USA; 4 Department of Pathology, St. Louis University School of Medicine, St. Louis, USA; 5 Forensic Pathology, Office of the Medical Examiner, St. Louis, USA

**Keywords:** color, cutaneous, dermatology, forensic, melanogenesis, postmortem, radiation, sunburn, tanning, ultraviolet

## Abstract

Postmortem tanning, previously referred to as either postmortem suntan or postmortem sunburn, presents as hyperpigmentation of sun-exposed uncovered skin. It most commonly occurs in decedents who remain in an environment in which the ambient temperature is either warm or hot; the areas of the corpse that are exposed to the sun develop hyperpigmentation. This postmortem change usually appears in the fresh (first) stage of decomposition. We describe three decedents who developed postmortem tanning. One of the women has an extensive severe darkening of her skin in the distribution where her skin was exposed to sunlight. The second woman had a localized band of pronounced tanning on her lower abdomen; associated early decomposition had occurred, including skin slippage and possible marbling of the superficial veins, at the location of the tanning. The third case, a young man, showed the concurrent presence of keratosis pilaris on his extensor arms that were affected by the tanning. In contrast to livor mortis, postmortem tanning occurs on the skin that is located on the non-dependent side of the body, those areas most apt to be exposed to sunlight. In living individuals, melanogenesis-associated tanning is usually an oxygen-dependent process. In decedents, blood flow and oxygen delivery to the skin that becomes darkened/hyperpigmented is not mandatory. Areas for future investigation include description of the histologic appearance of the tanned skin in comparison to adjacent, non-tanned skin, the incidence of postmortem tanning, the factors that may accelerate or decelerate the occurrence of postmortem tanning, and the possible correlation of the appearance of postmortem tanning as a feature that can be used to reveal information for estimating the postmortem interval.

## Introduction

Classic postmortem changes include algor mortis, livor mortis, and rigor mortis. These three changes are all part of the fresh (first) stage of decomposition (typically days one to two). The other stages of decomposition include the early decay or bloat (second) stage (days two to seven), the active decay (third) stage (days five to 13), the advanced decay (fourth) stage (days 10 to 23), and the dry remains (fifth) stage (days 18 to more than 90). The postmortem stages of decomposition may be used by forensic pathologists to estimate the postmortem interval [1-4].

A unique postmortem change includes postmortem tanning. Albiet, rarely described, postmortem tanning presents as darkening/hyperpigmentation of the sun-exposed areas of skin. As postmortem tanning is rarely documented, it may currently be considered to be a stage within the broader process of decomposition, rather than being an independent phenomenon. The incidence and pathogenesis of postmortem tanning remains to be established [4,5].

We present the clinical features of postmortem tanning in three decedents. Their manifestations of tanning after death provide additional insight into this phenomenon. The presence of postmortem tanning may be a clinical observation that has medicolegal implications since it might potentially be used in the estimation of the postmortem interval.

## Case presentation

Case 1

**Figure 1:**
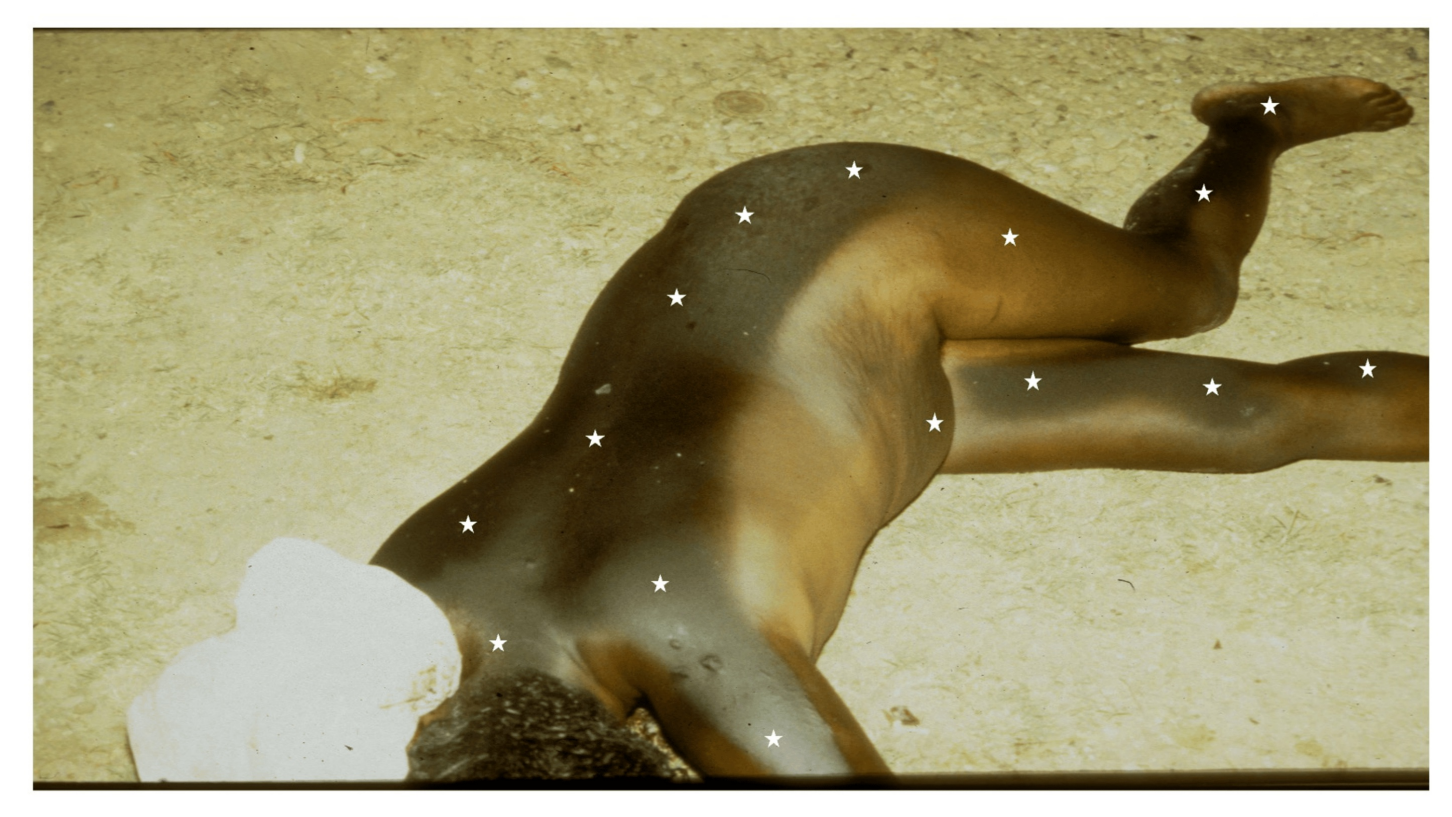
Severe postmortem tanning in a middle-aged Black woman A middle-aged Black woman who had died was discovered with extensive sun-related hyperpigmentation. There was marked darkening of the skin exposed to the sun on her left flank, upper back, left posterior arm, left hip, left lateral lower extremity and right medial leg show dark hyperpigmentation (white stars). The tanned areas that had been exposed to the sun since she was found lying on her right side. The figure has previously been published; however, details of the case have not been previously reported. Republished with permission from Springer Nature from Prahlow JA, Byard RW. Postmortem changes and time of death (Chapter 8). In: Prahlow JA, Byard RW, eds. Atlas of Forensic Pathology. New York, New York. Springer Humana Press; 2012, page 158 [4].

The body of a nude middle-aged Black woman, lying on her right flank, was discovered outdoors on a hot, sunny summer day, with extensive sun-related skin darkening/hyperpigmentation. There was marked darkening of the skin exposed to the sun on her left flank, upper back, left posterior arm, left hip, left lateral lower extremity, and right medial leg (Figure 1); less prominent tanning was observed in the areas of her upper left chest and abdomen in which the sun exposure had been partially blocked by clothing material. The figure has previously been published in a forensic pathology atlas with the following legend “Sun exposure following death can actually lead to dark discoloration of the exposed skin surface, a so-called ‘postmortem suntan’ or ‘sun burn’" [4].

She had been lying on her right side for several hours. Fixed livor mortis was present on the right side. 

Case 2

**Figure 2:**
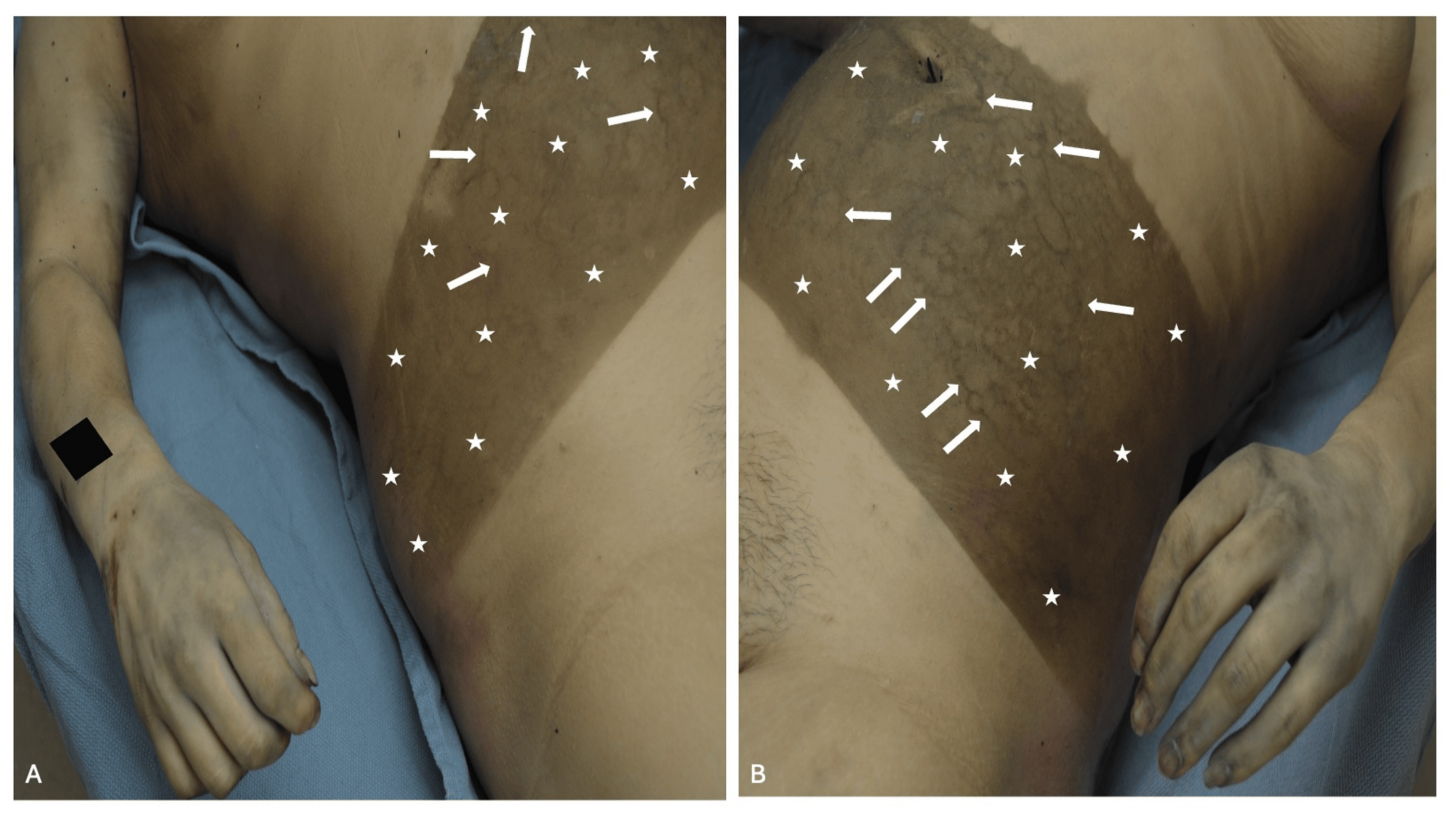
Postmortem tanning on the lower abdomen of a young Caucasian woman The hyperpigmentation from postmortem tanning on the right side (A) and left side (B) of the lower abdomen is sharply outlined within the area of sun-exposed skin that was not covered by clothing (white stars). In addition, skin dessication/slippage and early marbling of the superficial vessels which appears as darkening of the vessels is noted in the area of hyperpigmentation (white arrows). The black rectangle is covering a tattoo.

A Caucasian woman was discovered outdoors on a hot, sunny summer day. She had been lying on her back, and the bottom of her shirt had been removed from her pants, exposing her lower abdomen. Therefore, only a localized area of her abdomen had not been covered by her clothing. Examination showed prominent hyperpigmentation of her lower abdomen from above her umbilicus to the level of her suprapubic region (Figure 2). The skin in the area of darkening demonstrated early slippage/dessication with possible underlying marbling of the superficial vessels (consistent with the early decay and second stage of decomposition).

Case 3

**Figure 3:**
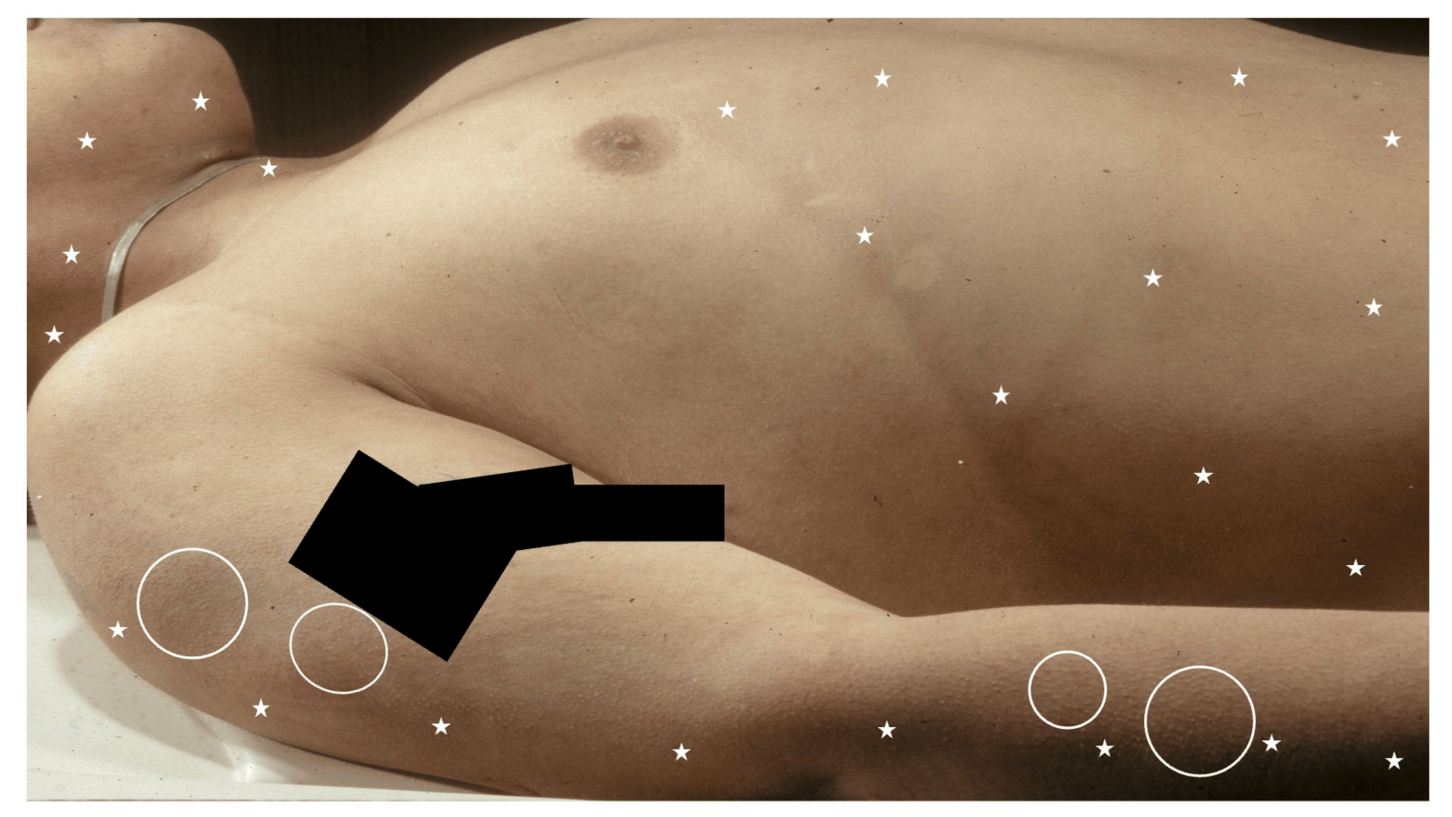
A young man with hyperpigmentation from postmortem tanning on sun-exposed skin Postmortem tanning is present on the right side of his neck, the lower chest (beneath the right areola), abdomen, and lateral right arm (white stars). Keratosis pilaris, presenting as small follicular papules, is also present in the area of hyperpigmentation (within white ovals). The black rectangles are covering a tattoo.

A young Caucasian man’s body was found outdoors on a warm, sunny summer day. He had been lying on his back, and his shirt had been partially removed. There was subtle, yet definite darkening/hyperpigmentation of the areas of his skin that had been exposed to the sun, with adjacent clothing-covered areas of skin demonstrating no darkening. The skin darkening was noted on the right side of his neck, the lower chest (beneath the right areola), abdomen, and lateral right arm (Figure 3). In addition, he had several small papules on the lateral arms, clinically consistent with keratosis pilaris; the darkening occurred on both the skin with and without papules.

## Discussion

Sun-exposure related skin darkening/hyperpigmentation of decedents was originally described within the forensic literature by Prahlow and Byard in 2012 [4]. They published a book that included the photograph of a female corpse that had developed profound hyperpigmentation in areas that had been exposed to the sun; the investigators referred to the observation as postmortem suntan or postmortem sunburn [4]. One of the authors had regularly observed this postmortem change while working in the state of Texas (in the United States of America), in decedents who were found outdoors and whose skin was not completely covered, typically on very sunny, hot days during the summer.

Subsequently, another group of researchers from North Dakota (in the United States of America) reported three decedents with similar skin discoloration of their sun-exposed skin in 2023. They described their observation as postmortem tanning. They commented that this was an “unusual postmortem event” and that “no literature citations and [only] a single image of this phenomenon in an atlas underscore an incomplete and patchy acceptance of this postmortem change [5].”

The three cases in this report provide some unique features regarding postmortem tanning (Table 3) [6,7]. The first woman (case one) had extensive sun-related skin darkening/hyperpigmentation. Importantly, livor mortis occurs on the dependent parts of the body irrespective of the exposed or unexposed part of the body, whereas tanning will occur only on the sun-exposed parts. The woman’s postmortem tanning was on her back and her left side; the livor mortis was on her right flank. This case confirms that postmortem tanning can affect individuals with a dark complexion.

**Table 1:** Characteristics of decedents with postmortem tanning Abbreviations: L, left; M, man; R, right. ^a^The Fitzpatrick classification of sun-reactivity skin types was originally described in 1975 and only included white-skinned persons. It was revised in 1976 to also include brown-skinned individuals and black-skinned individuals [6]. ^b^The colorimetric scale for skin of color was introduced in 2023. The classification scale includes five skin color types: type 1 (very light beige), type 2 (light brown), type 3 (medium brown), type 4 (dark brown), and type 5 (very dark brown) [7]. ^c^Fitzpatrick skin type VI has a skin color of unexposed skin of black. The erythema reaction is never burns. The tanning reaction is always tans [6]. ^d^A skin color type of 2 corresponds to a light brown color of skin [7]. ^e^Fitzpatrick skin type IV has a skin color of white. The erythema reaction is rarely burns. The tanning reaction is tan more than average (with ease) [6]. The classification of skin type 4 in this person is primarily based on the tanning reaction that was observed. ^f^Only individuals with skin of color are included in the colorimetric classification. The designation of skin of color excludes a person with white skin. Therefore, a person with white skin would have a skin color type 0 when being evaluated using the colorimetric classification scale for skin of color [7].

Case	Gender	Age	Skin color	Fitzpatrick classification^a^	Colorimetric classification^b^	Tanning location	Associated features
1	Woman	Middle-aged	Black	VI^c^	2^d^	L flank, L posterior arm, L hip, L lateral extremity, R upper leg, upper back	None
2	Woman	Young	White	IV^e^	0^f^	Lower abdomen	Marbling of the superficial vessels, skin desiccation, skin slippage
3	Man	Young	White	IV^e^	0^f^	Abdomen, R lateral arm, R lower chest, R side of neck	Keratosis pilaris

The second woman (case two) was found to be lying supine; her shirt had been raised above her umbilicus. Only a localized area of her lower abdomen had been exposed to the sun. She had changes of more advanced decomposition on the sun-exposed areas. These changes included the development of skin slippage and early marbling of the superficial vessels (which is usually observed in the early active, second stage of decomposition).

The rate of decomposition is particularly influenced by the ambient temperature [1-3]. Indeed, Weber et al. postulated that two of the decedents they reported had a delayed onset of the typical decompositional skin discoloration based on the hypothermia of the corpses [5]. We hypothesize that the localized accelerated decomposition in the second woman we described was caused by the direct sun exposure, resulting in an increase in temperature in the sun-exposed skin.

The young man (case three) had an underlying skin dermatosis (keratosis pilaris). The sun-related hyperpigmentation occurred not only in the normal-appearing skin but also on the skin affected by his underlying cutaneous condition. His circumstances demonstrate that postmortem tanning can occur concurrently on both disease-free skin and dermatosis-affected skin.

The mechanism of postmortem tanning remains to be determined. There are several mechanisms whereby solar radiation results in discoloration/pigmentation of the skin. Interactions between keratinocytes and melanocytes in the epidermis and/or hair follicles result in skin pigmentary responses; the responses are influenced by autocrine, endocrine, intracrine, nutritional, and paracrine factors [8].

Exposure to ultraviolet A radiation (in the wavelength range of 320 to 400 nanometers) in living individuals results in rapid darkening of melanin and immediate tanning. The total quantity of melanin is unchanged; however, the stored melanin is released from the melanocytes. The onset of immediate tanning within living subjects can be within minutes; it can persist for approximately one day [8,9]. Although speculative, perhaps similar mechanisms occur in postmortem skin when conditions are appropriate, resulting in postmortem tanning.

Exposure to ultraviolet B radiation (in the wavelength range of 280 to 320 nanometers) results in melanogenesis, with an increase in the production of melanin and delayed tanning. The tanning lasts much longer than the tanning resulting from ultraviolet A radiation. Experimentation demonstrated that erythema and pigment response to ultraviolet B radiation was not altered when oxygen was eliminated by stopping the blood flow to the area exposed to the ultraviolet B radiation [8,9]. This mechanism of skin tanning is probably less likely to be at play in postmortem skin, since normal metabolism ceases when death occurs; however, since it is possible to harvest and grow skin fibroblasts in culture several hours after death has occurred, it is theoretically possible for such a mechanism to be instrumental in the development of postmortem tanning.

Two of the decedents described by Weber et al. were found in settings where the ambient temperature was very cold; one died from hypothermia and was completely frozen, and the other had been exposed to cold winter January temperatures for 20 hours [5]. The researchers suggested that the decomposition of the bodies was delayed by the cold temperatures, and the enzyme processes that permitted melanogenesis were preserved until the bodies thawed and enabled the postmortem tanning to occur [5].

Similar to our patients, Weber et al.’s third patient died in a warm summer month of August [5]. Our patients were not exposed to abnormally cold environments; in contrast, they were more likely to be found in settings where the ambient temperature was elevated. Hence, the development of their tanning was likely to have been predominantly the result of a non-oxygen-dependent mechanism of melanogenesis and melanin release from melanocytes.

Histological and biochemical studies are inevitably needed to explain this discoloration. To date, no report exists that attempts to evaluate postmortem skin tanning histologically. Future investigation should incorporate pathological and biochemical studies in order to elucidate additional information about this phenomenon.

As the presented cases illustrate, skin tanning not only occurs during life but also after death, as long as conditions are appropriate. The phenomenon may be related to sunlight-initiated melanin release from melanocytes or even via transient melanogenesis. Postmortem tanning is possibly more commonly present than suggested by the current reports in the literature describing this phenomenon. Additional investigation is warranted to determine the incidence of postmortem tanning and what factors influence its formation. Future research should focus on whether the detection of postmortem tanning can be correlated with an estimation of the postmortem interval.

Photographs depicting the position of the body at the time of discovery will help investigators to understand the phenomenon of postmortem tanning. One final potential explanation for postmortem sun-tanning deserves mention. Ultimately, readily evident postmortem skin tanning may simply represent the very initial stages of decomposition as expressed on the skin surface, before definitive, more advanced stages of decomposition are evident.

Photographs of the deceased at the time of discovery of the corpse are important. It is well known within the forensic pathology community that, as the decomposition process ensues, the skin becomes darker. Such skin discoloration is so common that some forensic pathology textbooks show illustrative photographs depicting Caucasian individuals in fairly advanced states of decomposition having skin that is so dark that it would be easy to confuse the decedent for a Black person [10].

Another textbook makes note of the fact that variable decompositional changes of the skin of a given decedent can occur related to differing degrees of environmental exposure, including sunlight; with a photograph showing largely intact, non-decomposed skin of the lower body, and very dark, decomposing skin of the head and neck [11]. In select cases, in bodies exposed to intense sunlight, postmortem tanning may be one of the initial stages of decomposition changes. Alternatively, post-mortem skin tanning may represent advancing decompositional skin changes that outpace other typical features of decomposition. The fact that the second case presented in this report has associated skin slippage and early marbling in the area of skin hyperpigmentation, without any additional evidence of decomposition, suggests that this is exactly what is happening in this case.

## Conclusions

Postmortem tanning describes a change that may be observed in decedents whose skin is exposed to sunlight. The appearance of the tanning in the corpse demonstrates darkening/hyperpigmentation of the sun-exposed skin. The postmortem change typically appears in the first stage of decomposition, the fresh stage, during the period when the corpse develops algor mortis, livor mortis, and rigor mortis. However, in two decedents exposed to severely low ambient temperatures presented previously in the literature, the tanning was observed several days after death. Most decedents who develop postmortem tanning have been exposed to direct sunlight in locations with warm ambient temperatures. One woman had skin slippage and early marbling, which is typically observed in the early decay (second) stage of decomposition, in the distribution of the tanning; we speculate that the elevated temperature localized to the affected area may have accelerated the local decomposition of the body at that site. The exact pathogenesis of postmortem tanning has yet to be definitively established. We postulate not only the development of tanning-associated melanin release, but also melanogenesis. Postmortem melanogenesis, if present in such cases, is not restricted to an oxygen-dependent process, since blood flow and oxygen delivery to the skin essentially ceases when the heart stops. Future studies should evaluate the incidence of postmortem tanning, the factors that may accelerate or decelerate its occurrence, and the possible correlation of its appearance as an additional source of information that might be able to contribute to providing an estimate of the postmortem interval in decedents.
